# Cancer dormancy and criticality from a game theory perspective

**DOI:** 10.1186/s41236-018-0008-0

**Published:** 2018-01-22

**Authors:** Amy Wu, David Liao, Vlamimir Kirilin, Ke-Chih Lin, Gonzalo Torga, Junle Qu, Liyu Liu, James C. Sturm, Kenneth Pienta, Robert Austin

**Affiliations:** 1 Banter AI, 408 Florence St., Palo Alto CA, 94301 USA; 2 Department of Pathology, University of California at San Francisco, San Francisco, 94143 USA; 3 Department of Physics, Princeton University, Princeton, 08544 NJ USA; 4 Department of Electrical Engineering, Princeton University, Princeton, 08544 USA; 5 The Johns Hopkins Hospital, 1800 Orleans St., Baltimore MD, 21287 USA; 6 College of Optoelectronic Engineering, Shenzhen University, Shenzhen, 518060 China; 7 College of Physics, Chongqing University, Chongqing China, 400044 China

**Keywords:** Cancer, Dormancy, Game theory, Perturbations, Simulation

## Abstract

**Background:**

The physics of cancer dormancy, the time between initial cancer treatment and re-emergence after a protracted period, is a puzzle. Cancer cells interact with host cells via complex, non-linear population dynamics, which can lead to very non-intuitive but perhaps deterministic and understandable progression dynamics of cancer and dormancy.

**Results:**

We explore here the dynamics of host-cancer cell populations in the presence of (1) payoffs gradients and (2) perturbations due to cell migration.

**Conclusions:**

We determine to what extent the time-dependence of the populations can be quantitively understood in spite of the underlying complexity of the individual agents and model the phenomena of dormancy.

## Background

Dormancy is the relatively long period between treatment for cancer and the progression (return) and spreading of the cancer. After initial surgery and/or chemotherapy, the cancer apparently ceases to grow and is said to be in remission, or dormancy if the period is substantially longer than typical progression times for that cancer and treatment. Unfortunately often the cancer after this dormant period ends is resistant to the initial therapy that was used. We do not address the emergence of resistance here but rather the dynamics of dormancy and progression, although the emergence of resistance is a critical part of cancer progression (Han et al. 2016).

The main focus of this work in connecting cancer emergence and dormancy is the proposed phenomena of criticality in interacting cancer cell dynamics. Criticality has been used to describe many slow-driven, interaction-dominated, threshold dynamical systems (Jensen 1998) including evolution (Raup 1994) and morphogenesis (Krotov et al. 2014). Near the threshold of criticality strong amplification of fluctuations emerges in response to external perturbations (Sornette 2000). In a finite system exhibiting noncritical behavior, the distribution of systematic response to external perturbation can be characterized by the moments of mean and variance.. However, in critical systems, probability distributions of response follow power law decays, *P*(*s*)∼*s*^−*b*^. If the distribution has “thick tails”, that is with power-law coefficients *b*<3, then the mean and variance do not exist. In that case, external perturbation can lead to a response of any size (Yang 2004). We propose that dormancy and recurrence is a criticality problem, and use a game theoretical approach to analytically describe the phenomena.

## Methods

Simulations of Game Theory popualtion dynamics were run on a MacPro utilizing a 3.7 GHz Quad-Core Intel Xeon E5 processor. The coding was done using MaTLab 2016b.

## Results: population dynamics in interacting Cancer/Host cell populations

In order to characterize mixed population dynamics some sort of simple model is necessary, we have chosen game theory (Axelrod et al. 2006). Although game theory may ignore many critical details (Adami et al. 2016), it is a beginning step towards addressing criticality in cancer. A simple evolutionary game model which includes the influence of different cell types on each other involves coupled ordinary non-linear differential equations (Maynard Smith 1982; Durrett and Levin 1994). First, we assume that when we can break a heterogenous tumor up into *N* small subpopulations and each subpopulation*j* is locally homogeneous in 2 different cell types. The local population of cancer cells (*γ*_*j*_) and stromal cells (*η*_*j*_) within the *j*^*th*^ subpopulation can be described by the ordinary non-linear differential equations:

1 $$ \frac{d \gamma_{j}}{dt}=(A_{j}p_{\gamma j}+B_{j}p_{\eta j})\gamma_{j}   $$

2 $$ \frac{d \eta_{j}}{dt}=(C_{j}p_{\gamma j}+D_{j}p_{\eta j})\eta_{j}   $$

where $p_{\gamma j} = \frac {\gamma _{j}}{\gamma _{j} + \eta _{j}}$ and $p_{\eta _{j}} = \frac {\eta _{j}}{\eta _{j} + \gamma _{j}}$. The payoff coefficients, *A*_*j*_, *B*_*j*_, *C*_*j*_ and *D*_*j*_ have very transparent physical interpretations: they represent the result of pairwise interactions between cells in lattice *j*. Since *p*_*γj*_+*p*_*ηj*_=1, the dependence of the cancer cell fractional population *p*_*γj*_ can be written as:

3 $$ \frac{dp_{\gamma j}}{dt} = p_{\gamma j}(1 - p_{\gamma j}) \left [ (A_{j}-C_{j}) p_{\gamma j} + (B_{j}-D_{j}) (1 - p_{\gamma j}) \right ]   $$

There are two obvious fixed points in the flow of the fraction of *γ* cells versus time, $p^{*}_{\gamma } = 1, p^{*}_{\gamma } = 0$, these two fixed points simply represent an initially pure *γ* or *σ* population which cannot change in composition. However, in general there are four more principal end points for the progression of the tumor. Two of them are straightforward: (1) If (*A*_*j*_−*C*_*j*_)<0 and (*B*_*j*_−*D*_*j*_)<0, host cells *η* win over cancer cells *γ* (this is called prisoner’s dilemma in Game Theory jargon), in our case the cancer cells are out-competed by the host cells, perhaps by immunosurveilance or impaired vascularization amongst other reasons; (2) if (*A*_*j*_−*C*_*j*_)>0 and (*B*_*j*_−*D*_*j*_)>0, cancer cells *γ* win over host cells *η* (this is called harmony in Game Theory jargon, but alas here the “harmony” means that cancer cells out-compete the host cells and then recurrence emerges). In both the prisoners’s dilemma and harmony outcomes, at infinite time only one cell type remains.

There are two other fixed points with non-zero numbers of both *γ* cells and *η* cells which give rise to stationary values. The fraction of cancer cells *γ* at this fixed point is:

4 $$ \tilde{p}^{*}_{\gamma j}=\frac{1}{1-\frac{A_{j}-C_{j}}{B_{j}-D_{j}}}   $$

**Fig. 1:**
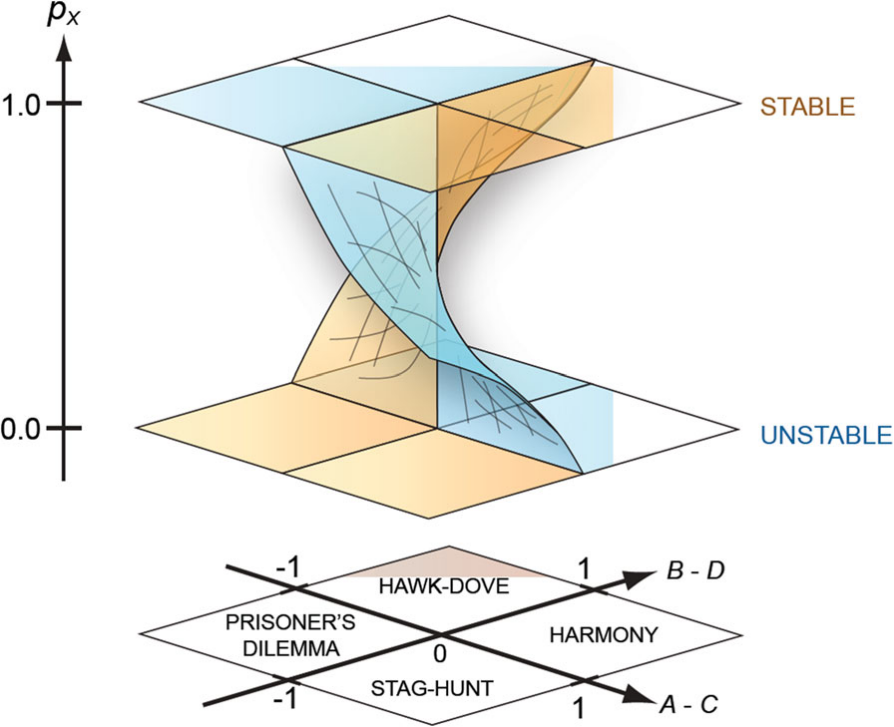
A 3D stability plot. The vertical axis encodes initial fraction of *γ* cells. The 2 planar axes are *A*−*C* and *B*−*D* parameters. The planar part shows the division into four quadrants which give rise to different scenarios for the fixed points. The blue surface represents the surfaces of unstable fixed points, and the brown surface represents the surfaces of stable fixed points

However, If *A*_*j*_−*C*_*j*_>0,*B*_*j*_−*D*_*j*_<0 this is an unstable fixed point and sensitive to perturbations. Since this point is unstable there is no residence time of the system at this point (this is known as a stag-hunt in Game Theory jargon). If (*A*_*j*_−*C*_*j*_)<0,(*B*_*j*_−*D*_*j*_)>0 the fixed point is stable. This case is called the hawk-dove game in Game Theory jargon, it is the only one allowing for stable coexistence of two populations. In terms of cancer population dynamics you would like to have coefficients such that optimally (*A*_*j*_−*C*_*j*_)<0,(*B*_*j*_−*D*_*j*_)<0, or at least (*A*_*j*_−*C*_*j*_)<0,(*B*_*j*_−*D*_*j*_)>0 so that the *γ*/*η* ratio does not diverge. Figure 1 presents graphically these population stability landscapes as a function of the pay-off matrix values.

**Fig. 2:**
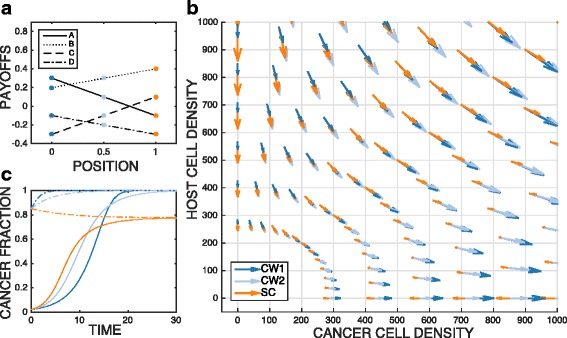
Payoffs Cancer wins (CW) to stable coexistence (SC) Blue: cancer wins (case 1), light blue: cancer wins (case 2), orange: stable coexisistence. **a** Payoffs vs. position. **b** The phase plane of cancer cell density vs. host cell density. The arrows indicate the fitness at given populations and payoffs. **c** Cancer fraction vs. time. Solid line: initial cancer fraction is 0.02. Dotted line: initial cancer fraction is 0.85

**Fig. 3:**
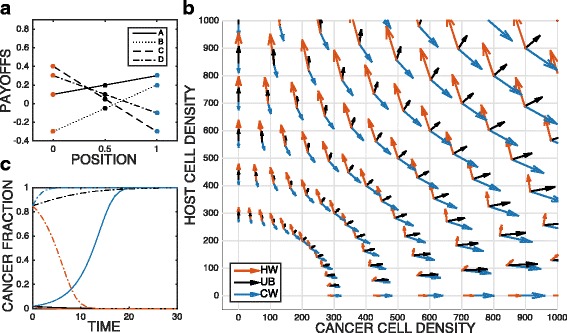
Unstable bifurcation (UB) to cancer wins (CW) Red: host wins, black: unstable bifurcation, blue: cancer wins. **a** Payoffs vs. position. **b** The phase plane of cancer cell density vs. host cell density. The arrows indicate the fitness at given populations and payoffs. **c** Cancer fraction vs. time. Solid line: initial cancer fraction is 0.02. Dotted line: initial cancer fraction is 0.85

It is a reasonable assumption that payoffs at neighboring subpopulations (*j* vs. *j*+1) change incrementally. Experimental evidence of payoff gradients has been demonstrated in a co-culture system of multiple myeloma and stromal cells within a linear drug gradient landscape (Wu et al. 2014). Here we will discuss two game transition scenarios across the landscapes of payoffs: (1) from cancer wins to stable coexistence, (2) from host wins, unstable bifurcation to cancer wins. First, as shown in Fig. 2a, the payoffs *A*,*B*,*C*,*D* are equal to 0.3,0.2,−0.3,−0.2 at position 0, and the payoffs change linearly to −0.1,0.4,0.1,−0.3 at position 1. Based on the payoffs coefficients at each position *j*, we can calculate which quadrant (the type of game) in Fig. 1 can represent the lattice *j*. The phase plane of cancer cell density vs. host cell density in Fig. 2b and the dynamics of cancer fraction in Fig. 2c shows cancer cells win at position 0 and 0.5 and coexist with host cells at position 1 independent of initial population densities. Secondly, in Fig. 3, the payoffs *A*,*B*,*C*,*D* are equal to 0.1,−0.3,0.4,0.1 at position 0, and the payoffs change linearly to 0.3,0.2,−0.3,−0.1 at position 1. Since we sweep through the unstable bifurcation zone in this case, whether cancer will win becomes sensitive to initial population fraction, as shown black lines in Fig. 3b and c.

Integration of Eq. (3) yields the equilibration time *τ* it takes for these fixed points to be approached:

5 $$ \tau= \int^{p_{fin}}_{p_{in}} \frac{dp}{p_{}(1-p_{})((A-C)p_{} + (B-D)(1-p_{}))}   $$

Near the stable and unstable fixed points *τ* diverges, the systems slows down and criticality may occur. In the case of the unstable fixed point (the stag-hunt), we can identify the dormancy period as the time spent in the vicinity of the unstable fixed point, and the recurrence of the cancer as the population moves away from the unstable fixed point. On the other hand, if the system has matrix elements such that we are in a hawk-dove quadrant the cancer while not “cured” (which is the prisoner’s dilemma end-point) but rather the cancer cells are in a stable equilibrium: chronically present but not life threatening, a region of permanent dormancy.

In the basic model presented above, we assumed each lattice *j* of tumor is a closed and homogenous region, no exchange of cells is involved. To gain more physiological relevance, we introduce cancer cell migration between lattices as a perturbation to the system. Such perturbation can also be a format of temporal varying payoffs, which are not discussed in this work.

At each time point, we assume cancer cells migrate with probabilities *m*^+^ (to the right neighboring lattice) and *m*^−^ (to the left neighboring lattice), and the migration of host cells are negligible. The equation of cancer cell density *γ* becomes:

6 $$ \gamma_{j}(t+1)=\gamma_{j}(t)+dt\left[A_{j}p_{\gamma j}(t)+B_{j}p_{\eta j}(t)\right]\gamma_{j}(t)+M_{j}(t)  $$

where migration term is:

7 $$ M_{j}(t)=-\left[m^{-}_{j}(t)+m^{+}_{j}(t)\right]C_{j}(t)+\left[m^{-}_{j+1}(t)C_{j+1}(t)+m^{+}_{j-1}(t)C_{j-1}(t)\right]  $$

**Fig. 4:**
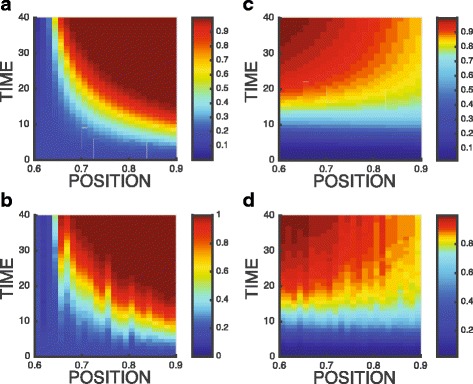
Cancer fraction vs. space and time: **a** and **c** no migration, **b** and **d** with migration. The initial cancer fraction is 0.02. Initial cancer fraction: 0.02. **a** and **b** Transitions from “host wins” to “unstable bifurcation” to “cancer wins.” **c** and **d** Transitions from “cancer wins” to “stable coexistence”

**Fig. 5:**
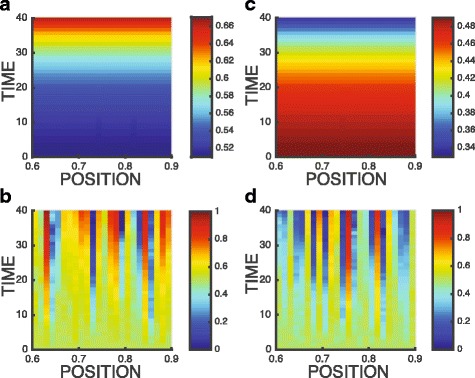
Cancer fraction vs. space and time in the vicinity of the unstable fix point (unstable bifurcation): **a** and **c** no migration, **b** and **d** with migration. **a** and **b** Initial cancer fraction: 0.51. **c** and **d** Initial cancer fraction: 0.49

We assume here weak migration: that is we assume *m*^+^ and *m*^−^ are normal random distributed with a mean equal 0 and standard deviation equal 0.03. That means 99.7*%* of simulated migration rates (percentage of cells migrate to neighboring lattices) is less than 9*%*. The effect of migration on spatio-temporal dynamics of cancer is shown in Figs. 4 and 5.

## Discussion

In Fig. 4a and b, the payoffs *A*,*B*,*C*,*D* are equal to 0.22,−0.1,−0.22,0.06, respectively at *x*=0.6 (where host wins, cancer fraction *p*→0), and the payoffs change linearly to 0.28,0.15,−0.23,−0.06 at *x*=0.9 (where cancer wins, cancer fraction *p*→1). As the position is close to the vicinity of bifurcation regime (*x*=0.65 in Fig. 4a), equilibration time to reach stationary state (*τ* in Eq. 5) increases. For example, *τ*=15 at *x*=0.9 as cancer fraction reaches stationary state *p*^∗^=1, while *τ*=30 at *x*=0.7. The migration of cancer cells is simulated in Fig. 4b, resulting a noisy pattern which is similar to Fig. 4a. In Fig. 4c and d, the payoffs *A*,*B*,*C*,*D* are equal to 0.08,0.32,−0.06,−0.22, respectively at *x*=0.6 (where cancer wins, cancer fraction *p*→1), and the payoffs change linearly to −0.08,0.38,0.06,−0.28 at *x*=0.9 (where host and cancer stably coexist, cancer fraction *p*→0.825). Likewise, same perturbation due to cancer cell migration as shown in Fig. 4d results in a noisy pattern similar to Fig. 4c.

Figure 5 demonstrate the effect of migration on spatio-temporal dynamics of cancer in a critical state near the vicinity of unstable equilibrium (*p*≈0.5). The payoffs *A*,*B*,*C*,*D* are equal to 0.14,−0.11,−0.01,0.04 at *x*=0.6, and the payoffs change linearly to 0.26,0.01,0.11,0.16 at *x*=0.9. First of all, the system slows down near the equilibrium. After 40 generations, cancer fraction slightly changes from 0.51 to 0.67 in Fig. 5a and from 0.49 to 0.33 Fig. 5c. After perturbation is introduced in Fig. 5b and c, we observe the amplification of fluctuation near the equilibrium. Within 40 generations, neighboring lattices can be dominated by either cancer *p*→1 or host cells *p*→0, exhibiting the critical behavior, a perturbation response of any size.

## Conclusions

Cancer dormancy is a slow-driven, interaction-dominated threshold system. Frequency of breast cancer recurrence rate indicates while non-metastatic instance follows exponential decay, metastatic instance may be a critical system which follows power law. We modeled cancer dormancy inspired by evolutionary game theory, and found that the payoffs modulated by microenvironmental factors (such as drug, oxygen, nutrients) dictate the dynamics of cancer cells vs. host cells (including stromal and immune cells). Perturbation (due to cancer cell migration) in the vicinity of equilibrium is associated with the loss of global stability and may lead to recurrence of metastatic cancer.

Much work remains to be done to map the landscape of the interaction coefficients and classify between stable regime and unstable regime, here we provide a first step towards identifying the dynamical signatures that could be used for prediction of emergence from dormancy.

We hope that this work will inspire more measurements, improve predictive power of cancer recurrence, and assist the control of cancer progression.
